# Source–sink nitrogen dynamics and their response to variable nitrogen levels during rice grain filling

**DOI:** 10.1186/s12870-026-09580-w

**Published:** 2026-07-20

**Authors:** Xiao-Li Huang, Yan Peng, Zhi-Jun Xu, Chao Huang, Yong-Liang Han, Jia-Shi Peng, Zhen-Ning Teng, Neng-Hui Ye, Shuan Meng, Jing Zhao

**Affiliations:** 1 Hunan Provincial Key Laboratory of Rice Stress Biology, Hunan Agricultural University, Changsha, 410128 China; 2 Yuelushan Laboratory, Changsha, 410128 China; 3 College of Agronomy, Hunan Agricultural University, Changsha, 410128 China; 4 College of Bioscience and Biotechnology, Hunan Agricultural University, Changsha, 410128 China; 5 College of Resources, Hunan Agricultural University, Changsha, 410128 China; 6 Hunan Key Laboratory of Economic Crops Genetic Improvement and Integrated Utilization, Hunan University of Science and Technology, Xiangtan, 411201 China

**Keywords:** Rice, Nitrogen, Grain filling, Source, Sink

## Abstract

**Background:**

Rice grains largely depend on nitrogen (N) remobilization from leaves. However, the dynamics of N species in source (leaf) and sink (grain) tissues and the effects of the external N supply remain unclear.

**Results:**

This study investigated the dynamic changes and metabolic characteristics of major N fractions in flag leaves and grains under high-nitrogen (HN) and low-nitrogen (LN) treatments during grain filling. The concentrations of grain nitrate-N (NO₃⁻-N) and amino acid-N (AAs-N) decreased, whereas ammonium-N (NH₄⁺-N) showed dynamic changes, leading to an increased proportion of inorganic N (IN). The total N (TN) in the grain was negatively correlated with NO₃⁻-N but positively correlated with NH₄⁺-N (LN: *r* = 0.75; HN: *r* = 0.92). In flag leaves, only NO₃⁻-N increased. Despite similar overall trends, the magnitudes of these changes varied among the N treatments. The grain TN was predominantly supported by AAs-N: under HN, AAs-N accounted for 74.1–50.0%, which consistently exceeded IN (25.9–49.9%); in contrast, under LN at the middle-to-late stages, IN (50.2–69.7%) exceeded AAs-N (49.8–30.3%). HN strengthened the positive correlation between grain NH₄⁺-N and TN, whereas LN intensified the correlations among the leaf N fractions. LN accelerated TN and AAs-N degradation and promoted NO₃⁻-N accumulation and N remobilization in leaves. Furthermore, LN upregulated grain N-assimilating enzymes and leaf NiR, whereas HN maintained relatively high leaf NR and GS. Transcriptomic analysis revealed that hormone signals were involved in regulating rice N metabolism.

**Conclusions:**

During rice grain filling, NO₃⁻-N, NH₄⁺-N, and AAs-N in grains and leaves dynamically changed and were differentially regulated by enzyme activity and gene expression, ultimately clarifying source‒sink nitrogen dynamics for optimized nitrogen management.

**Supplementary Information:**

The online version contains supplementary material available at 10.1186/s12870-026-09580-w.

## Background

Nitrogen (N) is one of the most critical nutrients for rice growth. It participates in morphological development and physiological metabolism, ultimately determining both yield and quality [1, 2]. N utilization is governed by integrated processes, including N metabolism, transport, and redistribution throughout the growth cycle [1, 3].

During the early growth stages, the N required by rice is acquired primarily from the external environment through root uptake. Rice roots mainly take up inorganic N in the forms of ammonium (NH₄⁺) and nitrate (NO₃⁻) while also utilizing organic forms such as amino acids and polypeptides [4, 5]. The majority of NO₃⁻ and part of the NH₄⁺ absorbed by the roots are loaded into the xylem and translocated to the shoot [5, 6]. Aside from a small fraction that is stored, most of this N enters the assimilation pathway. Specifically, NO₃⁻ is reduced to NH₄⁺ via the sequential action of nitrate reductase (NR) and nitrite reductase (NiR); the resulting NH₄⁺ is then incorporated into amino acids through the GS/GOGAT cycle [7]. In addition, glutamate dehydrogenase (GDH) also participates in N metabolism [7].

Later in the growth cycle, especially during the grain-filling stage, the N needed by the plant comes from continuous root N uptake as well as from the remobilization of N from vegetative organs to grains [8, 9]. This remobilization is the dominant process that governs grain yield. In both rice and wheat, most of the N accumulated in the grains originates from the remobilization of N that was previously stored in vegetative tissues [8, 10]. As the primary source of N redistribution, leaves contribute the majority of N accumulated in grains during grain filling [8, 11, 12].

Leaf N remobilization primarily occurs in the form of small organic N molecules such as amino acids [4, 13]. These organic N compounds are generated mainly from the degradation of N-containing macromolecules in leaves via autophagy or vacuolar proteases and are subsequently transported by specific transporters [9, 14]. Consequently, defects in autophagy or loss of function of related transporter genes impair N remobilization and lead to reduced yield [15, 16]. In addition, nitrate is also a major form of remobilized N [17]. Genetic engineering strategies aimed at enhancing the remobilization of leaf nitrate to grains have been shown to effectively increase rice yield [9].

The grain-filling stage is widely recognized as the most critical period for determining rice yield. During this phase, N demand in the grain is derived primarily from leaf N remobilization. However, the specific patterns of accumulation and transformation of different N components within the leaf “sources” and grain “sinks”—as well as their underlying relationships—remain poorly understood. Furthermore, the relative contributions of these dynamic changes to N translocation between leaves and grains have yet to be elucidated. To address these knowledge gaps, we implemented two distinct N application levels to analyze and compare the physiological characteristics of N utilization in both leaves and grains during the grain-filling stage.

## Materials and methods

### Plant materials and growth conditions

The experiment was carried out at Hunan Agricultural University (Changsha, China). The soil was a paddy soil with the following properties: organic matter content of 30.41 g·kg^− 1^, total N content of 1.81 g·kg^− 1^, total P content of 0.54 g·kg^− 1^, total K content of 8.93 g·kg^− 1^, available N content of 112.73 mg·kg^− 1^, available P content of 13.48 mg·kg^− 1^ and available K content of 122.73 mg·kg^− 1^. Two nitrogen (N) rates were applied: a high‑N treatment (HN, 180 kg N ha⁻¹) and a low‑N treatment (LN, 60 kg N ha⁻¹).

The rice cultivar Nipponbare (*Oryza sativa* L. subsp. *japonica*) was used as the experimental material. Seeds were sown on 8 June 2021, transplanted on 29 June, and harvested on 7 September. N fertilizer, in the form of urea, was applied in three split doses at the basal, tillering, and panicle initiation stages at a ratio of 5:3:2 (w/w/w). Potassium fertilizer (K₂O, 144 kg·ha⁻¹) was applied in two splits at the basal and panicle initiation stages at a 6:4 (w/w) ratio. Phosphorus fertilizer (P₂O₅, 60 kg·ha⁻¹) was applied as a basal dose. Standard management practices, including conventional irrigation, were followed throughout the growth cycle.

Panicles that displayed a uniform growth stage and tiller position were selected and marked after panicle excision. The flag leaves and grains of the marked panicles were harvested on the day of flowering and preserved as 0 days after flowering (DAF) reference samples. Subsequent time‑point collections were performed every five days (5, 10, 15, 20, 25, and 30 DAF). Each sample was immediately frozen in liquid nitrogen and stored at − 80 °C until further processing. At maturity, key agronomic traits, including plant height, tiller number, grain yield per plant, and grain number per panicle, were recorded.

### Determination of Total Nitrogen (TN) concentrations in grains and flag leaves

Similar to the approach used by Li et al. [18], as detailed below. Harvested rice grains and leaf tissues were dried to constant weight, after which they were ground into a fine powder. The TN concentration of the samples was then determined using a carbon–nitrogen elemental analyzer.

### Analysis of Nitrate Nitrogen (NO_3_^−^-N) concentrations in grains and flag leaves

For the extraction of NO_3_^−^, approximately 0.08 g of fresh sample ground in liquid nitrogen was weighed into a 2 mL centrifuge tube. After the addition of 800 µL of 0.2 M HCl, the mixture was homogenized by vortexing and placed on ice. The NO_3_^−^ was extracted by agitation at 100 rpm for 2 h at 4 °C, followed by centrifugation at 12,000 ×g for 15 min at 4 °C. The supernatant was collected and recentrifuged for 1–2 min; this supernatant was subsequently kept on ice until analysis.

For sample determination, 10 µL of the sample extract was added to a centrifuge tube, followed by the addition of 40 µL of 5% (w/v) salicylic acid in concentrated sulfuric acid. After incubation at room temperature for 20 min, 950 µL of 2 M NaOH was added, and the mixture was thoroughly mixed by vortexing until the yellow complex fully developed and until the precipitate completely dissolved. Once the mixture cooled to room temperature, it was briefly centrifuged, and the absorbance was measured at 410 nm. The NO_3_^−^-N concentration was subsequently calculated via a linear regression equation derived from the standard curve generated from a KNO_3_ solution and normalized to the sample fresh weight to determine the final NO_3_^−^-N concentration.

### Determination of the ammonium Nitrogen (NH_4_^+^-N) concentrations in grains and flag leaves

For the extraction of NH_4_^+^, approximately 0.08 g of fresh sample, ground to a fine powder in liquid nitrogen, was weighed into a 2 mL centrifuge tube. After the addition of 800 µL of 0.2 M HCl, the mixture was homogenized by vortexing and subsequently allowed to stand on ice. The NH_4_^+^ was extracted by agitation at 100 rpm for 2 h at 4 °C, followed by centrifugation at 12,000 ×g for 15 min at 4 °C.

For sample determination, a master mixture was prepared by premixing ultrapure water, 15% (v/v) NaOCl, and 6.25 M NaOH. The reaction was initiated by sequentially adding 40 µL of the sample, 20 µL of phenol reagent, and 140 µL of the master mixture. The mixture was homogenized by pipetting three times, sealed in a plastic bag, and incubated at 37 °C for 60 min. The absorbance was measured at 620 nm. The NH_4_^+^-N concentration was subsequently calculated via the linear regression equation derived from the standard curve generated from the (NH_4_)_2_SO_4_ solution and normalized to the sample fresh weight to determine the final NH_4_^+^-N concentration.

### Analysis of free Amino Nitrogen (AAs-N) concentrations in grains and flag leaves

Approximately 0.07 g of fresh sample, ground to a fine powder in liquid nitrogen, was weighed into a 2 mL centrifuge tube and mixed thoroughly with 800 µL of 80% (v/v) ethanol. The mixture was incubated in an 80 °C water bath for 30 min, followed by centrifugation at 12,000 ×g for 15 min at 4 °C. A 400 µL aliquot of the supernatant was concentrated to dryness using a vacuum rotary evaporator. The residue was subsequently reconstituted in 400 µL of 0.02 M HCl, and the resulting extract was kept on ice until amino acid analysis.

For sample determination, 150 µL of the prepared extract was mixed with 1.85 mL of pH 5.4 buffer, 3.0 mL of ninhydrin reagent, and 0.1 mL of 0.3% (w/v) ascorbic acid in a 25 mL stoppered glass tube. The reagents were added sequentially and mixed thoroughly before being heated in a boiling water bath for 15 min. After cooling to room temperature, the reaction mixture was diluted to a final volume of 20 mL with 50% (v/v) ethanol.

A 200 µL volume of the resulting solution was transferred to a 96-well microplate, and the absorbance was measured at 580 nm. The free AAs-N concentration in each sample was calculated using the linear regression equation derived from the standard curve established with leucine.

### Measurement of enzyme activities related to nitrogen and amino acid metabolism

The grain and flag leaf samples, which were previously stored at − 80 °C, were transferred to precooled mortar and rapidly pulverized into a fine powder, approximately 0.1 g of which was weighed for subsequent analysis.

The activities of enzymes involved in primary N assimilation—specifically nitrate reductase (NR; kit AKNM001M) and nitrite reductase (NiR; kit AKNM004M-100 S)—as well as those associated with amino acid metabolism—including glutamine synthetase (GS; kit AKAM008M), glutamate dehydrogenase (GDH; kit AKAM005M), and NADH-dependent glutamate synthase (NADH-GOGAT; kit AKAM009M)—were determined using commercial assay kits (Boxbio, Beijing, China). All procedures were performed following the manufacturer’s instructions, and the enzymatic activities were expressed as units per gram of fresh weight (U g⁻¹ FW).

### RNA sequencing and transcriptomic analysis

To evaluate the molecular responses during grain filling, grains and flag leaves were collected at 10 days after flowering (DAF). For each tissue, three independent biological replicates were prepared, with each replicate consisting of pooled samples from multiple individual plants to minimize biological variation. Total RNA extraction, library construction, and sequencing were performed by OE Biotech Co., Ltd. (Shanghai, China). The raw sequencing data were processed for quality control, and subsequent bioinformatics analyses—including differential expression analysis, Gene Ontology (GO) enrichment, and Kyoto Encyclopedia of Genes and Genomes (KEGG) pathway mapping—were performed using the OE Cloud tools.

### Statistical analysis

The data were subjected to statistical analysis using *Student’s t tes*t (for two-group comparisons) or *one-way analysis of variance (ANOVA)* followed by Duncan’s multiple range test (for multiple comparisons). Statistical significance was defined at *p* < 0.05 and *p* < 0.01, denoted by * and **, respectively. Pearson correlation coefficients were calculated to evaluate the relationships between parameters. All figures and statistical illustrations were generated using GraphPad Prism version 10.4.0 (GraphPad Software, San Diego, CA, USA).

## Results

### Performance of major agronomic traits under different nitrogen levels

**Fig. 1:**
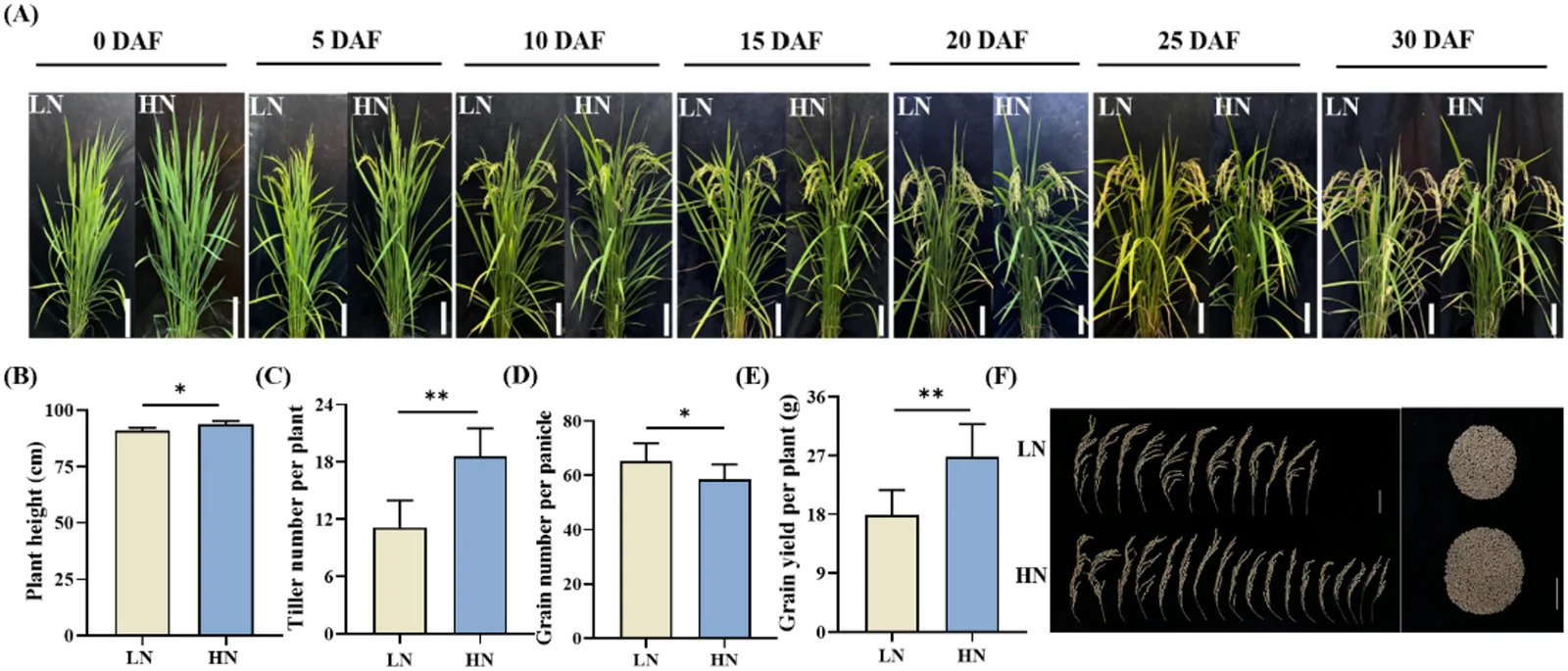
Phenotypic performance and major agronomic traits of rice under different N levels. **A** Plant phenotypes during the grain-filling stage. **B** Plant height. **C** Tiller number per plant. **D** Grain number per panicle. **E** Grain yield per plant. **F** Representative photographs of individual rice plants. Scale bars: (**A**) = 10 cm; (**F**) = 5 cm. The values in (**B**–**F**) represent the means ± SDs (*n* = 8). LN, low nitrogen treatment; HN, high nitrogen treatment. Asterisks (* and **) denote significant differences at the *p* < 0.05 and *p* < 0.01 levels, respectively

The phenotypic performance of rice at the post-flowering stage varied significantly under the different N treatments (Fig. 1A). Analysis of agronomic traits at harvest revealed that plant height was significantly greater under HN than under LN (Fig. 1B). Similarly, the tiller number per plant was significantly greater under HN than under LN (Fig. 1C). Statistical analysis of the number of grains per panicle revealed that it was significantly greater under LN than under HN (Fig. 1D). However, the grain yield per plant was still significantly greater under HN than under LN (Figs. 1E–F). These results demonstrate that the two N treatments were effective and suitable for analyzing N utilization in rice leaves and grains, as well as for assessing differences across various N levels.

### N accumulation and dynamic characteristics of rice grains

**Fig. 2:**
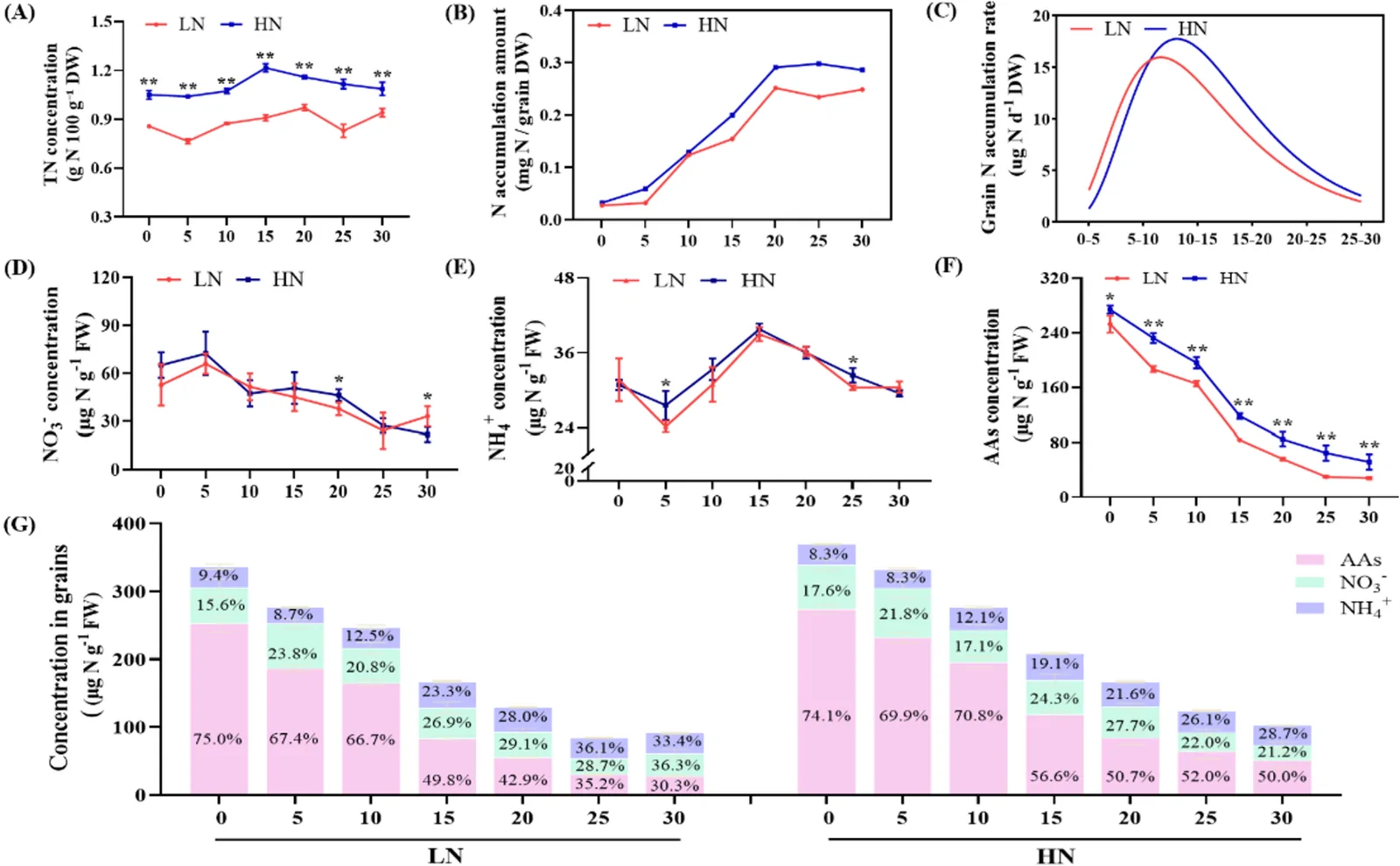
Accumulation and dynamic changes of nitrogen and its components in rice grains. **A** TN concentration. **B** N accumulation per grain. **C** Average N accumulation rate per grain. **D** NO_3_^−^-N concentration. **E** NH_4_^+^-N concentration. **F** AAs-N concentration. **G** Stacked proportions of different N components. The values in (**A**) represent the means ± SDs (*n* = 3); the values in (**D**–**F**) represent the means ± SDs (*n* = 4). The numbers 0, 5, 10, 15, 20, 25, and 30 in (**A**–**F**) denote days after flowering (DAF). Asterisks (* and **) denote significant differences at the *p* < 0.05 and *p* < 0.01 levels, respectively

During grain filling, the total nitrogen (TN) concentration in rice grains increased under both HN and LN conditions, with values consistently higher under HN than under LN. The peak TN concentration occurred 15 days after flowering (DAF) under HN but was delayed to 20 DAF under LN (Fig. 2A). During the reproductive growth period, grain N accumulation increased from 5 to 20 DAF before stabilizing, with consistently higher levels under HN than under LN (Fig. 2B). The TN accumulation rate followed an inverted-U pattern across the two treatments, peaking earlier under LN than under HN (Fig. 2C). The NO_3_^−^ -N concentration decreased continuously under both treatments, with a steeper decline under LN from 5 to 25 DAF (Fig. 2D). The NH_4_^+^-N concentration peaked at 15 DAF, displaying a decreasing–increasing–decreasing pattern in both treatments (Fig. 2E). The AAs-N concentration in the grains decreased throughout the period of grain filling and remained consistently greater under HN than under LN at all time points (Fig. 2F). Under both treatments, the proportion of AAs-N to TN decreased throughout grain filling, starting at 75.0% (LN) and reaching 74.1% (HN) at 0 DAF. In contrast, the NO_3_^−^-N and NH_4_^+^-N proportions increased overall, peaking under LN at 36.3% (NO_3_^−^-N, 30 DAF) and 36.1% (NH_4_^+^-N, 25 DAF), but at lower levels under HN (27.7% NO_3_^−^-N at 20 DAF; 28.7% NH_4_^+^-N at 30 DAF). Under LN, NO_3_^−^-N and NH_4_^+^-N exceeded AAs-N during late grain filling (25–30 DAF), whereas under HN, AAs-N remained relatively high throughout (Fig. 2G).

**Fig. 3:**
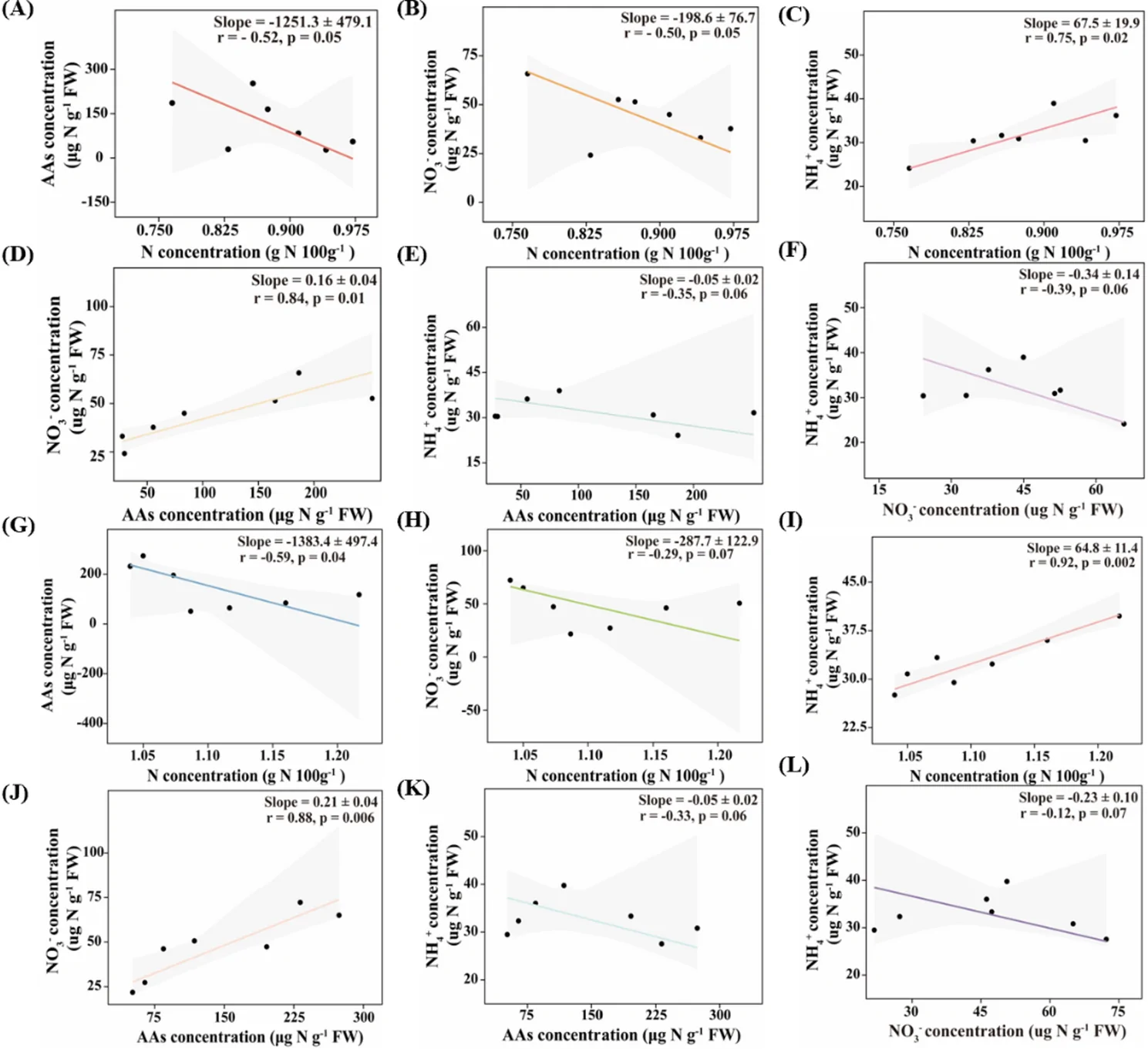
Correlations between different N components in rice grains during the grain-filling stage. **A**–**F** Correlation analysis of N components in grains under LN conditions: (**A**) TN and AAs-N; (**B**) TN and NO_3_^−^-N; (**C**) TN and NH_4_^+^-N; (**D**) AAs-N and NO_3_^−^-N; (**E**) AAs-N and NH_4_^+^-N; (**F**) NO_3_^−^-N and NH_4_^+^-N. **G**–**L** Correlation analysis of N components in grains under HN conditions: (**G**) TN and AAs-N; (**H**) TN and NO_3_^−^-N; (**I**) TN and NH_4_^+^-N; (**J**) AAs-N and NO_3_^−^-N; (**K**) AAs-N and NH_4_^+^-N; (**L**) NO_3_^−^-N and NH_4_^+^-N. Shaded areas represent the 95% confidence intervals. The values represent the means ± SDs (*n* = 3 for the TN concentration; *n* = 4 for the NO_3_^−^-N, NH_4_^+^-N and AAs-N concentrations). *r* denotes the Pearson correlation coefficient

Correlation analysis was conducted on the N components of the rice grains during the grain-filling stage (Fig. 3). Pearson correlation analysis revealed that TN was negatively correlated with AAs-N under both treatments (*r* = − 0.52 to − 0.59) and with NO_3_^−^-N (stronger under LN: *r* = − 0.50 vs. HN: *r* = − 0.29). In contrast, TN was strongly positively correlated with NH_4_^+^-N, which increased from *r* = 0.75 (LN) to *r* = 0.92 (HN). AAs-N and NO_3_^−^-N were positively correlated regardless of the N level (*r* = 0.88 and 0.84, respectively), whereas both were negatively correlated with NH_4_^+^-N (*r* = − 0.35 to − 0.33). The NO_3_^−^-N and NH_4_^+^-N correlations weakened substantially under HN (*r* = − 0.12).

### Nitrogen concentration and dynamic changes in flag leaves

**Fig. 4:**
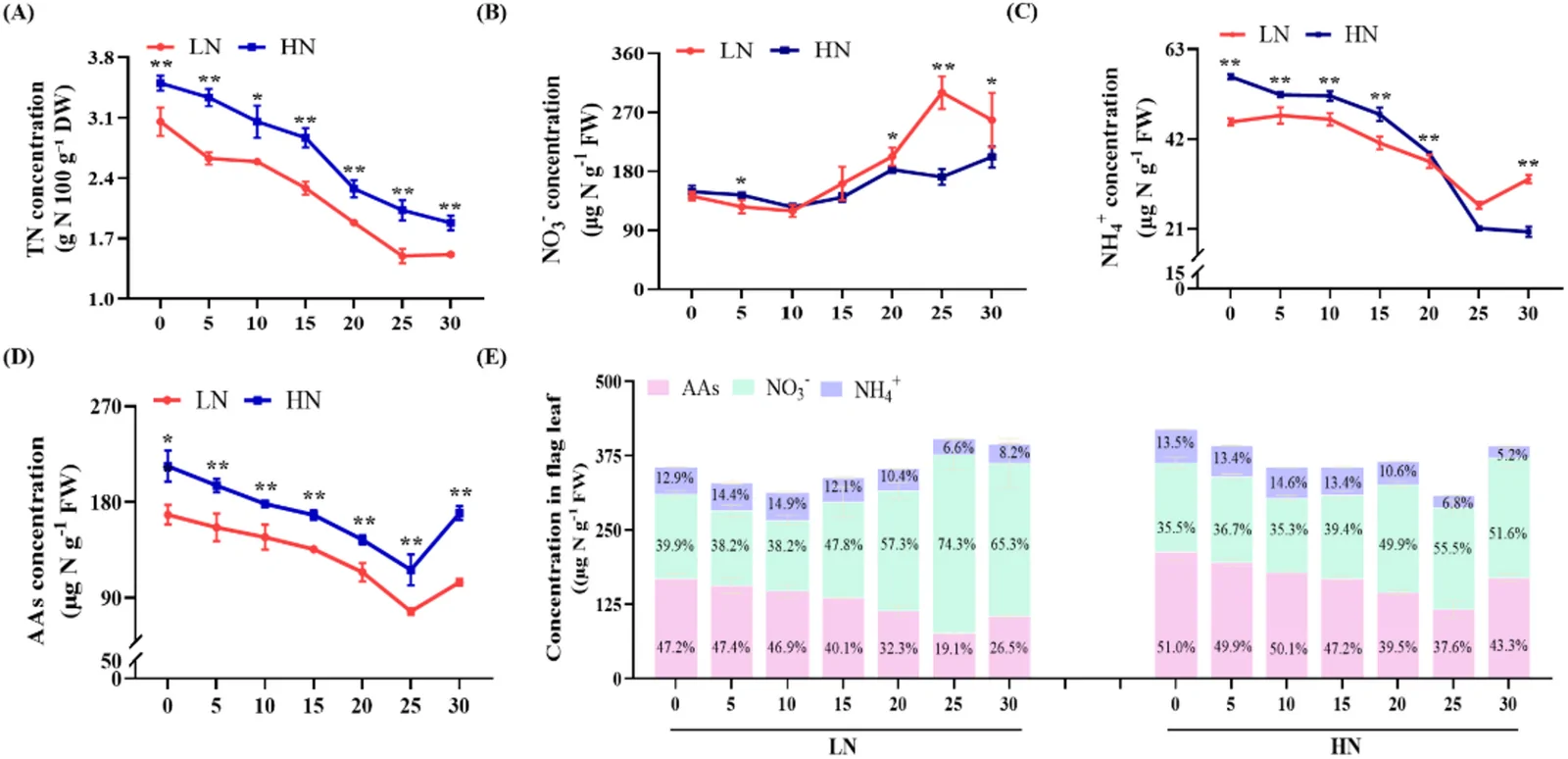
Stacked bar charts of N components in rice flag leaves during the grain-filling stage under different N levels. **A** TN concentration. **B** NO_3_^−^-N concentration. **C** NH_4_^+^-N concentration. **D** AAs-N concentration. **E** Proportional composition of different N components. The values in (**A**) represent the means ± SDs (*n* = 3); the values in (**B**–**D**) represent the means ± SDs (*n* = 4). The numbers 0, 5, 10, 15, 20, 25, and 30 in (**A**–**F**) denote the DAF. Asterisks (* and **) denote significant differences at the *p* < 0.05 and *p* < 0.01 levels, respectively

The flag leaf TN, NH_4_^+^-N, and AAs-N concentrations generally decreased throughout grain filling, whereas the NO_3_^−^-N concentration increased under both the HN and LN treatments (Fig. 4). The TN and AAs-N concentrations remained significantly greater under HN than under LN, yet their reductions were more pronounced under LN (Fig. 4A and D). For NO_3_^−^-N, the increase under LN substantially exceeded that under HN, surpassing HN levels from 10 DAF onward (Fig. 4B). The NH_4_^+^-N concentration initially decreased but then increased after 25 DAF, specifically under LN, reaching levels higher than those under HN by 30 DAF (Fig. 4C). Under both the HN and LN treatments, the AAs-N and NH_4_^+^-N proportions in flag leaves overall decreased, whereas NO_3_^−^-N increased (Fig. 4E). Under LN, AAs-N and NH_4_^+^-N reached minima at 25 DAF (19.1% and 6.6%), whereas NO_3_^−^-N peaked at 74.3%; under HN, the AAs-N minimum was 37.6%, and the NO_3_^−^-N maximum was 55.5%, which was also at 25 DAF. Unlike that under LN, NH_4_^+^-N continued to decrease under HN to 5.2% at 30 DAF. Temporally, AAs-N decreased from 0 to 25 DAF before rebounding; NO_3_^−^-N rose sharply from 15 to 30 DAF and then declined; and NH_4_^+^-N decreased from 15 to 25 DAF, rebounding late under LN but continuing to decline under HN.

Analysis of N remobilization revealed that the N transport amount (NTA) was lower under LN conditions than under HN conditions (1.54 vs. 1.62). These findings indicate that the actual N output from flag leaves to grains after heading was greater under HN than under LN. However, the N transport efficiency (NTE) was greater under LN (0.50) than under HN (0.46), suggesting that while the absolute N output was lower, the N remobilization capacity of flag leaves was greater under LN conditions (Fig. S1).

**Fig. 5:**
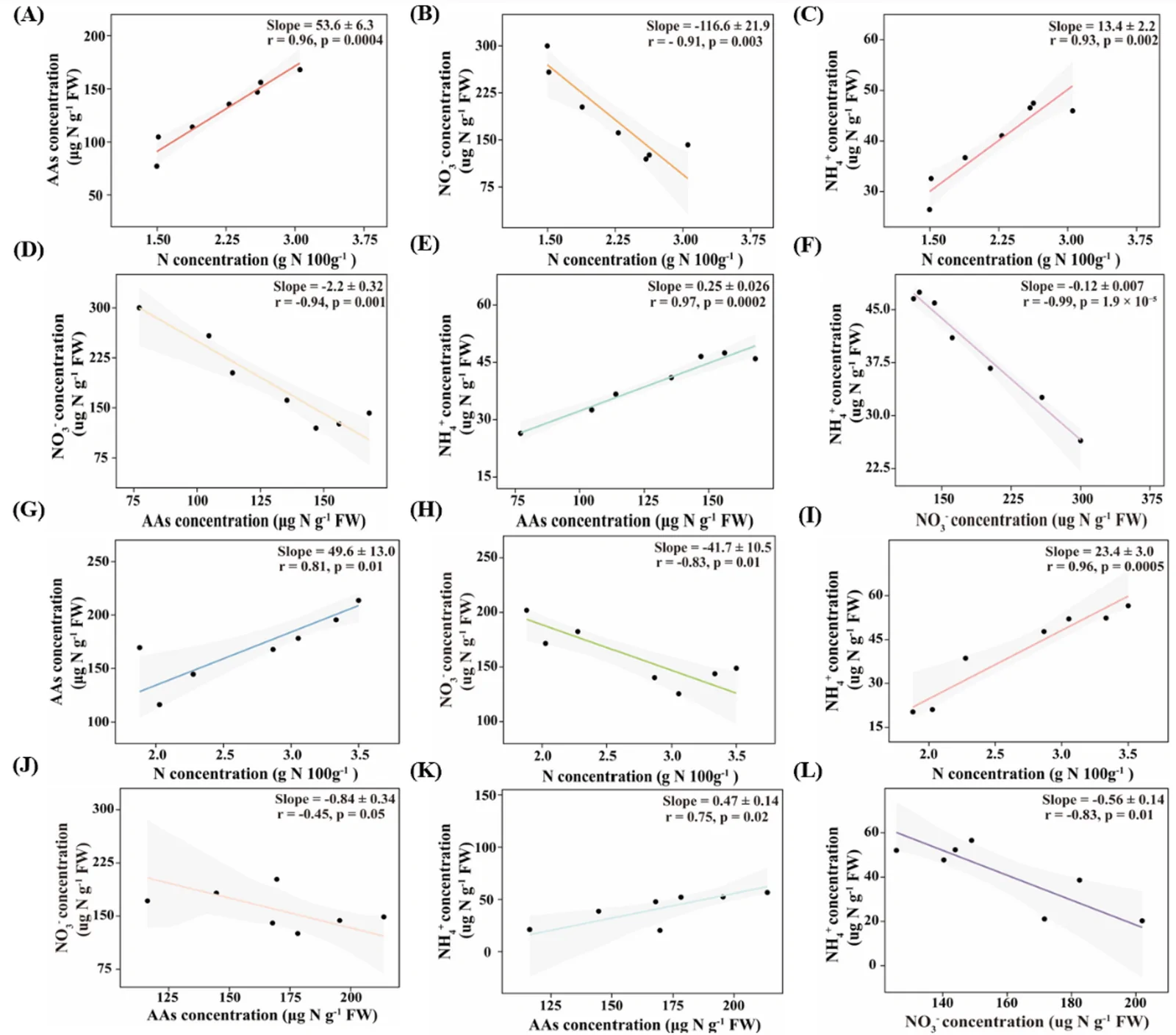
Correlation analysis of N components in rice flag leaves during the grain-filling stage under different N levels. **A**-**F** Correlations between N components under LN conditions: (**A**) TN and AAs-N; (**B**) TN and NO_3_^-^-N; (**C**) TN and NH_4_^+^-N; (**D**) AAs-N and NO_3_^-^-N; (**E**) AAs-N and NH_4_^+^-N; (**F**) NO_3_^-^-N and NH_4_^+^-N; (**G**–**L**) Correlations between N components under HN conditions: (**G**) TN and AAs-N; (**H**) TN and NO_3_^-^-N; (**I**) TN and NH_4_^+^-N; (**J**) AAs-N and NO_3_^-^-N; (**K**) AAs-N and NH_4_^+^-N; (**L**) NO_3_^-^-N and NH_4_^+^-N. The shaded areas represent the 95% confidence intervals. The values for the TN concentration represent the means ± SDs (*n* = 3), whereas the values for NO_3_^-^-N, NH_4_^+^-N and AAs-N represent the means ± SDs (*n* = 4). *r* denotes the Pearson correlation coefficient

Pearson correlation analysis of rice flag leaf N components during grain filling (Fig. 5) revealed that TN was strongly positively correlated with AAs-N (LN: *r* = 0.96, HN: *r* = 0.81) and NH_4_^+^-N (LN: *r* = 0.93, HN: *r* = 0.96) but negatively correlated with NO_3_^−^-N (LN: *r* = − 0.91, HN: *r* = − 0.83). Among the N fractions, AAs-N and NH_4_^+^-N exhibited extremely strong positive correlations under LN (*r* = 0.97) and weakened under HN (*r* = 0.75); AAs-N and NO_3_^−^-N were negatively correlated (LN: *r* = − 0.94, HN: *r* = − 0.45); NO_3_^−^-N and NH_4_^+^-N showed strong negative correlations under LN (*r* = − 0.99) and negative correlations under HN (*r* = − 0.83). Most correlations were stronger under LN than under HN, suggesting more tightly coordinated N metabolism under N limitation.

### Physiological basis and transcriptional responses of nitrogen utilization in rice grains and flag leaves under varying nitrogen levels

**Fig. 6:**
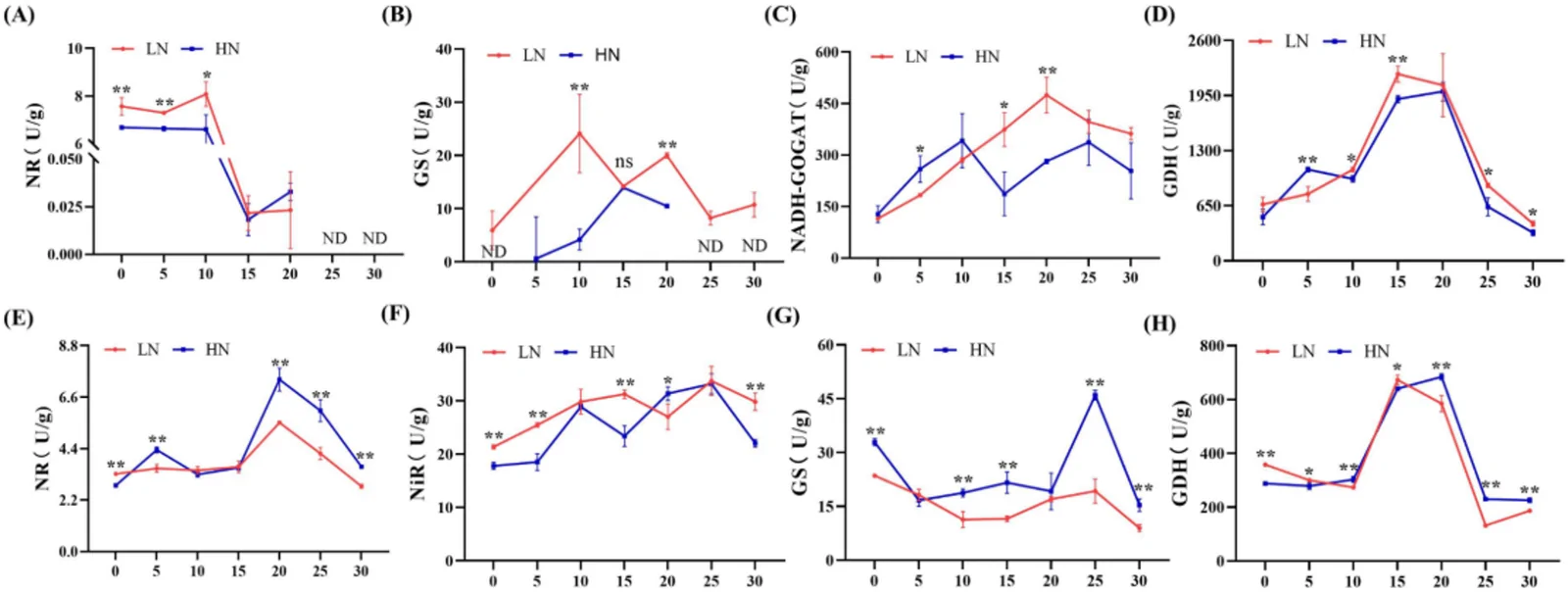
Activities of key N metabolism enzymes in rice grains and flag leaves during the grain-filling stage under different N levels. (A–D) Enzyme activities in grains: (**A**) nitrate reductase (NR); (**B**) glutamine synthetase (GS); (**C**) NADH-dependent glutamate synthase (NADH-GOGAT); and (**D**) glutamate dehydrogenase (GDH). **E**–**H** Enzyme activities in flag leaves: (**E**) NR; (**F**) nitrite reductase (NiR); (**G**) GS; and (**H**) GDH. The numbers 0, 5, 10, 15, 20, 25, and 30 denote the DAF. The values represent the means ± SDs (*n* = 3). Asterisks (* and**) denote significant differences at the *p* < 0.05 and *p* < 0.01 levels, respectively

Grain N metabolism enzyme activities were analyzed during grain filling (Fig. 6). Nitrate reductase (NR) activity declined overall under both treatments but more sharply under LN; however, NR activity was significantly greater under LN during early grain filling (0–10 DAF) (Fig. 6A). Glutamine synthetase (GS) and NADH-dependent glutamate synthase (NADH-GOGAT) activities both increased early in grain filling (Fig. 6B–C). GS activity remained consistently greater under LN than under HN throughout. In contrast, the level of NADH-GOGAT initially increased under HN (0–10 DAF) but surpassed that under HN at approximately 15 DAF under LN. The activity of glutamate dehydrogenase (GDH) tended to increase in a parabolic manner, with greater activity under LN during middle-to-late grain filling (Fig. 6D).

The flag leaf enzyme activities during grain filling are presented in Fig. 6E–H. NR activity peaked at 20 DAF in both treatments but was significantly greater under HN than under LN during the mid-to-late grain-filling stage (15–30 DAF). Nitrite reductase (NiR) activity peaked at 25 DAF but then decreased in both treatments (Fig. 6F). With the exception of approximately 20 DAF, the LN treatment had significantly greater effects than did the HN treatment throughout the period. GS activity showed an overall downward trend (Fig. 6G) and remained consistently greater under HN than under LN; however, the peak timing differed between the treatments (earlier under LN versus approximately 25 DAF under HN). GDH activity exhibited an arch-shaped pattern in both treatments (Fig. 6H), with the activity of LN peaking earlier (approximately 15 DAF) than that of HN (approximately 20 DAF). Specifically, GDH activity was greater under LN from 0 to 5 DAF, whereas that under HN exceeded that under LN from 20 to 30 DAF.

**Fig. 7:**
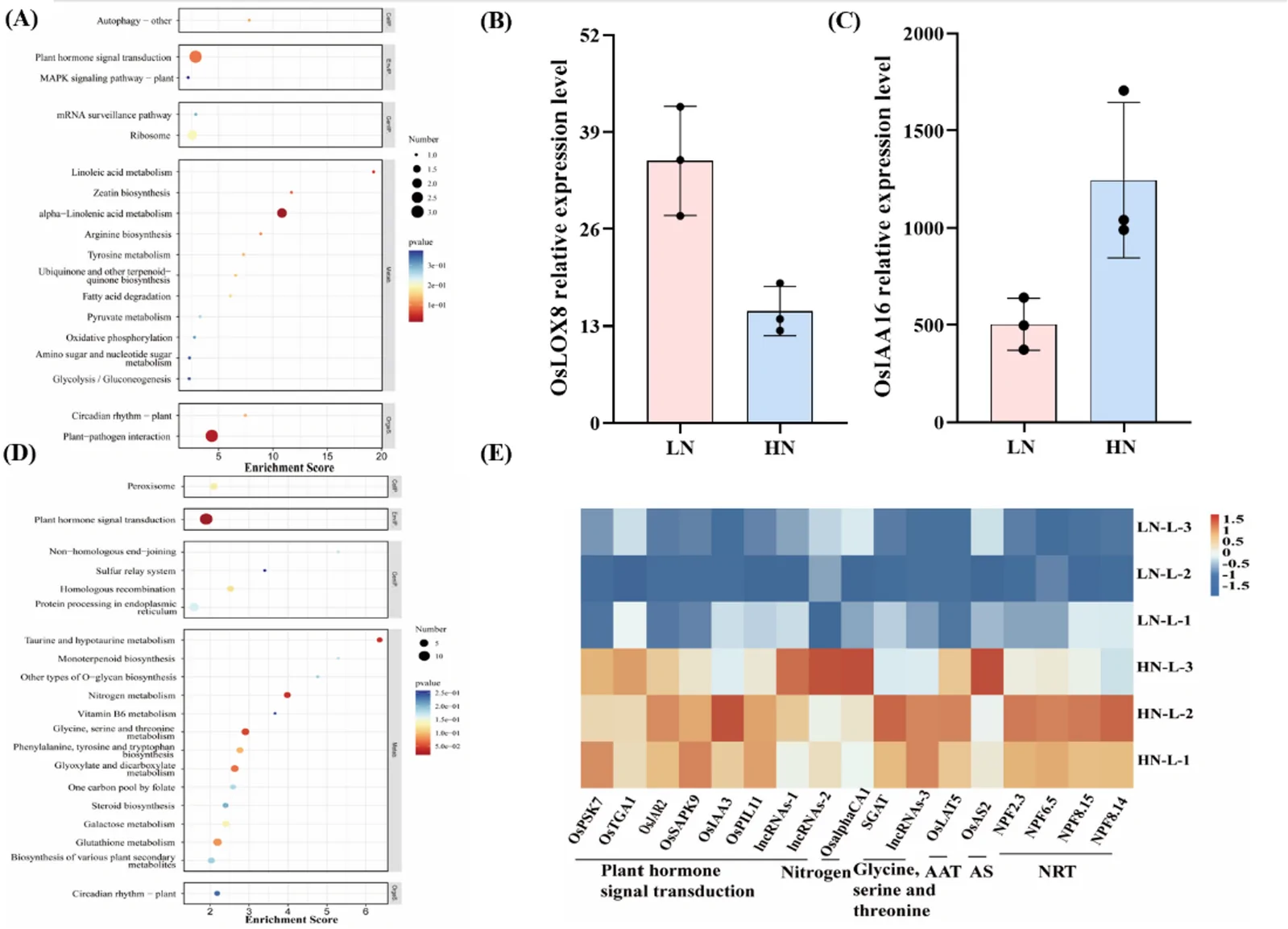
Transcriptome analysis of rice grains and flag leaves during the key grain-filling period under different N levels. **A**–**C** Analysis of grains: (**A**) KEGG enrichment pathways under HN and LN conditions; (**B**) expression levels (FPKM values) of *OsLOX8*; (**C**) expression levels (FPKM values) of *OsIAA16*. **D**–**E** Analysis of flag leaves: (**D**) KEGG enrichment pathways under HN and LN conditions; (**E**) heatmap of key DEGs within enriched pathways and N-related genes. . In the enrichment bubble plots, the dot color represents the *p* value, while the dot size indicates the number of DEGs associated with each pathway. The values in (B–C) represent the means ± SD (*n* = 3)

To further elucidate the response mechanisms of rice to N availability, transcriptome analysis was conducted using RNA-seq (RNA sequencing) on grains and flag leaves sampled at 10 DAF under both HN and LN conditions. In grains, a total of 127 differentially expressed genes (DEGs) were identified, comprising 67 up-regulated and 60 down-regulated genes (Figs. S2A, B). KEGG enrichment analysis revealed that the DEGs were significantly enriched in key pathways, including α-linolenic acid metabolism” and “plant-pathogen interaction” (Fig. 7A). Specifically, five DEGs were identified within these two pathways, including *OsLOX8* (Os08g0509100) and *OsAdh3* (Os11g0210600), which are involved in α-linolenic acid metabolism. Furthermore, in the “plant hormone signal transduction” pathway—which presented the highest degree of DEG enrichment—three genes were identified: *OsIAA16* (Os05g0186900), *OsTGA1* (Os05g0443900), and *OsPR1a* (Os07g0129200). These candidate genes potentially participate in the regulation of N utilization during grain development.

Transcriptomic analysis of flag leaves during the grain-filling stage revealed a total of 776 differentially expressed genes (DEGs), comprising 387 up-regulated and 389 down-regulated genes (Figs. S2C, D). KEGG pathway enrichment analysis revealed that DEGs were significantly enriched in several major pathways, including “plant hormone signal transduction,” “taurine and hypotaurine metabolism,” “nitrogen metabolism,” and “glycine, serine, and threonine metabolism” (Fig. 7D). Further screening of these enriched pathways identified 17 key DEGs (Fig. 7E). Specifically, eight genes were identified in the “plant hormone signal transduction” pathway: *OsPSK7* (Os11g0557000), *OsTGA1* (Os05g0443900), *OsJAR2* (Os01g0221100), *OsSAPK9* (Os12g0586100), *OsIAA3* (Os01g0231000), *OsPIL11* (Os12g0610200), *lncRNAs-1* (Os01t0923750-00), and *lncRNAs-*2 (Os03t0268750-00). Additionally, one DEG involved in nitrogen metabolism (*OsαCA1*, Os02g0533300) and two DEGs involved in glycine, serine, and threonine metabolism *(SGAT*, Os08g0502700; *lncRNAs-3*, Os10t0516150-00) were identified. Furthermore, six genes were localized to N transport pathways, including *OsLAT5* (Os03g0576900), *OsAS2* (Os06g0265000), *NPF2.*3 (Os12g0638300), *NPF6.5* (Os10g0554200), *NPF8.15* (Os10g0111300), and *NPF8.14* (Os10g0370700). These candidate genes potentially modulate N-utilization responses in leaves during the grain-filling process.

## Discussion

N is an essential macronutrient for rice, and its availability determines both grain yield and quality [1, 2, 19]. Consistent with these findings, we found that HN levels significantly increased grain yield. Further analysis revealed that N availability markedly regulates rice tillering; specifically, HN enhances yield by promoting tiller formation and increasing the number of effective panicles, which aligns with previous findings [20]. While the regulatory mechanisms by which N regulates tillering—including N transport, metabolic regulation, and hormonal signaling—have been extensively documented [20–22], the processes occurring during the grain-filling stage remain less understood. During this stage, N in the grains is derived primarily from leaf N translocation [13]. Although the transport of N to grains and its subsequent metabolism are crucial for grain development and N accumulation [23], the detailed N utilization characteristics and their underlying regulatory mechanisms during this specific process remain to be fully elucidated.

Our results revealed that the TN concentration in rice grains increased during the grain-filling stage, with levels under HN conditions being significantly greater than those under LN conditions (Fig. 2). Higher free AAs-N levels and a greater capacity for amino acid incorporation in developing grains are positively associated with increased protein accumulation in mature rice grains, indicating that amino acid availability during grain filling is a key determinant [24]. Under HN, the proportion of AAs-N remained higher than that of inorganic N (the sum of NO_3_^−^-N and NH_4_^+^-N) throughout the grain-filling period. However, under LN, the proportion of inorganic N exceeded that of AAs-N during the mid-to-late stages (Fig. 2G). During grain filling, dynamic changes in component concentrations highlight AAs-N as the primary factor driving these differences. The grain AAs-N concentration was significantly greater under HN than under LN. In contrast, the IN concentration did not differ significantly between HN and LN. Furthermore, the AAs-N concentration decreased progressively throughout grain filling, indicating that the TN concentration in rice grains is driven primarily by the AAs-N fraction. This finding also implies that while rice maintains abundant AAs-N under sufficient N, N deficiency limits the supply needed to meet increasing demands in later development, resulting in a lower AAs-N ratio. This pattern aligns with that reported by Caputo and Barneix [25], who reported that the rate of amino acid exudation from leaves to the phloem depends on N availability: a sufficient N supply promotes exudation to the phloem, whereas N deficiency leads to N allocation toward leaf protein synthesis. On the basis of this evidence, we propose that the LN supply may reduce the amount of amino acid transport to the grain. This, in turn, restricts amino acid conversion to storage proteins, thereby resulting in decreased amino acid-derived N and relatively increased inorganic N proportions in the grain. NTE analysis revealed that flag leaves presented greater N remobilization capacity under LN conditions (Fig. S1), even though the absolute nitrogen output was lower. Studies have shown that increasing N application from 0 to 360 kg N ha⁻² shifts rice N utilization, promoting early N storage and continuous later uptake under high nitrogen [26]. This strategy reduces N remobilization efficiency [26, 27], a finding that is consistent with our observation of lower NTE under HN than under LN.

Correlation analysis (Fig. 3) revealed that across N levels, TN was negatively correlated with AAs-N, whereas NO_3_^−^-N and AAs-N were positively correlated. NH_4_^+^-N and TN were also positively correlated. Notably, the r value between grain NH_4_^+^-N and TN reached 0.92 under HN conditions. Nitrate reductase (NR) is a key enzyme for nitrogen acquisition by plants [28], whereas the glutamine synthetase/glutamate synthase (GS/GOGAT) cycle represents the primary pathway for converting inorganic NH_4_^+^-N into glutamine (Gln) and glutamate (Glu) [7, 29]. Under LN conditions, NR activity was significantly greater than that under HN activity in the early grain-filling stage. Similarly, GS activity in grains increased under LN, whereas NADH-GOGAT and GDH activities increased during the middle-to-late grain-filling stages (Figs. 6A–D). These results suggest that under N deficiency, rice adapts to the environment by increasing the activity of key N metabolism enzymes, thereby strengthening internal N assimilation, transport, and remobilization processes to increase NUE.

Flag leaf TN and AAs-N both declined more rapidly under both the LN and the HN treatments during grain filling, indicating increased N remobilization efficiency from leaves to grains under N deficiency. In rapeseed, LN treatment similarly induces leaf senescence, leading to the degradation of proteins into AAs, which are subsequently exported via transporters [30]. NO_3_^−^-N in flag leaves increased during grain filling, with a significantly greater increase under LN than under HN. This may result from accelerated catabolism and reduced anabolism under N deficiency, promoting N translocation to grains (Fig. 4). Correlation analysis revealed that flag leaf TN was positively correlated with AAs-N and NH_4_^+^-N but negatively correlated with NO_3_^−^-N under both HN and LN conditions. AAs-N and NH_4_^+^-N were positively correlated, whereas NO_3_^−^-N was inversely correlated with both (Fig. 5).

In terms of enzyme activity, NR activity in flag leaves was greater under HN than under LN, in contrast to the trend observed in grains. Notably, NiR activity was greater under LN than under HN, except at 20 DAF. Unlike the elevated GS activity in grains under LN, flag leaves presented greater GS activity under HN, which is consistent with previous reports showing that leaf GS activity increases with N availability [31]. This may be attributed to LN stress reducing the overall N concentration, soluble protein levels, and activity of key metabolic enzymes, thereby inhibiting photosynthetic capacity and weakening N assimilation. The resulting decrease in NR activity leads to an insufficient NO_3_^-^-N supply, which in turn reduces GS, potentially minimizing N consumption (Figs. 6E–H).

The grain-filling stage in rice is a critical period during which hormonal signaling and N remobilization coordinately regulate yield formation. Previous studies have demonstrated that hormones such as abscisic acid (ABA) and cytokinin (CK) influence key yield traits—including grain filling, the seed setting rate, and thousand-grain weight—by modulating leaf senescence and N transport efficiency [32]. Indole-3-acetic acid (IAA) modulates nitrate transporter gene expression via auxin response factors to regulate nitrogen uptake efficiency in rice [33]. *OsHI-LOX* is involved in jasmonic acid (JA) biosynthesis and affects resistance to herbivorous insects in rice [34]. Through transcriptomic analysis of grains and flag leaves during the peak grain-filling period (Fig. 7), we found that *OsLOX8* was up-regulated while *OsIAA16* was down-regulated in grains under LN conditions. Consistent with findings in *Arabidopsis*, where JA and IAA signaling exhibit antagonistic interactions to balance plant growth and defense responses [35], our results suggest a similar trade-off in rice grains. Moreover, the rice *NRT* family plays a pivotal role in N absorption, transport, and redistribution [36]. In flag leaves, we identified several nitrate transporter genes (*NRTs*) whose expression were up-regulated under HN conditions, suggesting that HN induces shoot N transport to optimize whole-plant N allocation. For example, *OsNRT1.1B* (*OsNPF6.5*), which is expressed primarily in roots and up-regulated by N supply, has been shown to increase N uptake and adaptability to HN environments [37]. However, in the present study, the synchronized up-regulation of multiple NRT family members in the flag leaves further demonstrates a comprehensive activation of the shoot N transport system under HN conditions.

## Conclusions

Our study systematically investigated source–sink nitrogen dynamics and their response to variable nitrogen levels during rice grain filling. During rice grain filling, NO₃⁻-N, NH₄⁺-N, and AAs-N in grains and flag leaves dynamically changed. While the overall trends of these nitrogen components throughout grain filling were similar across different nitrogen levels, their magnitudes of change and absolute concentrations varied. These variations were differentially regulated by enzyme activity and gene expression, ultimately providing insights into source‒sink nitrogen dynamics for optimized nitrogen management.

## Supplementary Information

Supplementary Material 1.

## Data Availability

The datasets generated and analyzed during the current study have been deposited in the Genome Sequence Archive in National Genomics Data Center (PRJCA064595) that are publicly accessible at https://ngdc.cncb.ac.cn/bioproject/browse/PRJCA064595.

## Abbreviation

ABA: Abscisic acid
AMT: Ammonium transporter
AAs-N: Amino acid nitrogen
ANOVA: Analysis of variance
DAF: Days after flowering
DEGs: Differentially expressed genes
GDH: Glutamate dehydrogenase
Gln: Glutamine
Glu: Glutamate
GOGAT: Glutamate synthase
GO: Gene Ontology
IAA: Indole-3-acetic acid
IN: Inorganic nitrogen (nitrate and ammonium)
JA: Jasmonic acid
KEGG: Kyoto Encyclopedia of Genes and Genomes
NADH-GOGAT: NADH-dependent glutamate synthase
NH_4_^+^-N: Ammonium nitrogen
NO_3_^-^-N: Nitrate nitrogen
NiR: Nitrite reductase
NR: Nitrate reductase
NRT: Nitrate transporter
NTA: Nitrogen transport amount
NTE: Nitrogen transport efficiency
RNA-seq: RNA sequencing
TN: Total nitrogen
