# Supplementary material for: Source–sink nitrogen dynamics and their response to variable nitrogen levels during rice grain filling
Source: BMC Plant Biol. 2026 Jul 20;26:1419. doi: 10.1186/s12870-026-09580-w (PMC13487986; doi:10.1186/s12870-026-09580-w)
Supplement: Supplementary file 1 [file 12870_2026_9580_MOESM1_ESM.docx]

**Figure. S1.** Nitrogen accumulation (NTA) and nitrogen translocation efficiency (NTE). **(A)** Nitrogen translocation amount (NTA) under low and high nitrogen conditions; **(B)** Nitrogen translocation efficiency under low and high nitrogen conditions. Data in the figures were calculated from nitrogen contents in flag leaves at the heading and maturity stages; values in (A–B) represent the mean ± SD (*n* = 4).

**Figure S2.** Transcriptomic analysis of rice grains and flag leaves at key grain-filling stages under different nitrogen levels. **(A)** Number of differentially expressed genes (DEGs) in grains under different nitrogen levels; **(B)** Clustering heatmap of DEGs in grains under different nitrogen levels; **(C)** Number of DEGs in flag leaves under different nitrogen levels; **(D)** Clustering heatmap of DEGs in flag leaves under different nitrogen levels. In the hierarchical clustering heatmaps, filtering criteria were set as q-value < 0.05 and |log₂FC| > 1.
